# Beyond correlation: functional OPTO-MAgnetic Integration Concept (OPTOMAIC) to reveal the brain-wide signature of local neuronal signals-of-interest

**DOI:** 10.1117/1.NPh.9.3.032213

**Published:** 2022-07-06

**Authors:** Dirk Cleppien, Felipe Aedo-Jury, Albrecht Stroh

**Affiliations:** a Leibniz Institute for Resilience Research, Mainz, Germany; b Institute of Pathophysiology, University Medical Center of the Johannes Gutenberg University, Mainz, Germany

**Keywords:** fMRI, optical calcium recordings, blood oxygen level-dependent response onset determination, line-scanning fMRI, optical fiber-based fMRI analysis pipeline

## Abstract

**Significance:**

Due to the vascular origin of the fMRI signal, the spatiotemporally precise interpretation of the blood oxygen level-dependent (BOLD) response as brain-wide correlate of neuronal activity is limited. Optical fiber-based neuronal calcium recordings provide a specific and temporally highly resolved signal yet lacking brain-wide coverage. The cross-modal integration of both modalities holds the potential for unique synergies.

**Aim:**

The OPTO-MAgnetic Integration Concept (OPTOMAIC) extracts the very fraction of the BOLD response that reacts to optically recorded neuronal signals-of-interest.

**Approach and Results:**

First, OPTOMAIC identifies the trials containing neuronal signal-of-interest (SoI) in the optical recordings. The long duration of the BOLD response is considered by calculating and thresholding neuronal interevent intervals. The resulting optical regression vector is probed for a positive BOLD response with single-event and single-voxel resolution, generating a BOLD response matrix containing only those events and voxels with both a neuronal SoI and a positive fMRI signal increase. Last, the onset of the BOLD response is being quantified, representing the section of the BOLD response most reliably reporting at least components of the neuronal signal.

**Conclusions:**

The seven OPTOMAIC steps result in a brain-wide BOLD signature reflecting the underlying neuronal SoI with utmost cross-modal integration depth and taking full advantage of the specific strengths of each method.

## 1 Introduction

In functional magnetic resonance imaging (fMRI) experiments, the blood oxygen level-dependent (BOLD) contrast in a measured voxel is dependent on regional variations in blood flow, blood volume, or blood oxygen concentration.^1^^,^^2^ These contrast changes can be measured and evaluated brain-wide. Through the mechanism of neurovascular coupling, neuronal activity as the signal-of-interest (SoI) impacts these hemodynamic parameters in form of a specific BOLD response with a duration of several seconds. However, the SoI cannot be unambiguously determined in the observed fMRI signal trace because of the temporally extended BOLD response^3^ and its bias due to overlying neurometabolic processes that occur even in the absence of any neuronal spiking activity.^4^ In addition, vascular processes, such as respiration and heartbeat, and signal noise complicate signal interpretation. This limits the spatiotemporal interpretation of the fMRI signal as a correlate of neuronal SoI.

In contrast, optical fiber-based calcium recordings allow direct measurement of neuronal SoI in a local brain volume.^5^^,^^6^ For this purpose, cells are either stained with a fluorescent calcium indicator such as Oregon-Green BAPTA-1 or transduced with genetically encoded fluorescent calcium indicators such as GCaMP6.^7^^,^^8^ Subsequently, the calcium transients of at least 30 simultaneously firing neurons^9^ can be recorded in a bulk-manner without single-cell depiction as the SoI with high temporal resolution. Monitoring of neuronal activity is thereby limited to the detected signal from the fluorescent cell volume.

Cross-modal integration of BOLD fMRI with simultaneously measured optical fiber-based calcium recordings now opens the possibility of identifying the brain-wide measured hemodynamic BOLD response of the local optically measured SoI of a reference volume. The cross-modal approach is possible because the detection of the calcium transients is not disturbed by the strong magnetic field of the MR system,^10^^–^^18^ while the implanted optical fiber does not cause signal artifacts due to field distortions in the fMRI measurements.

In our own study, Schwalm et al.^19^ discovered that slow-wave activity characterized by slowly alternating periods of neuronal silence and activity measured as SoI of a localized brain volume in anesthetized rats can lead to a transcortical hemodynamic BOLD response. For this purpose, periods of neuronal slow-wave activity were interpreted as neuronal events, transformed into a regression vector for event-based signal analysis of fMRI signal traces, and then evaluated.

Schlegel et al.^10^ showed the feasibility of the cross-modal approach also in mice. Chen et al.^20^ were able to link subcortical neuronal activity obtained with calcium recordings to the hippocampal fMRI signal, e.g., showing distinct spatiotemporal features of hemodynamic responses of the hippocampal vasculature. To implant the fiber reliable into small subcortical regions like the corpus callosum^21^ they also invented an MR-guided robotic arm approach during the surgery.^22^ Wang et al.^23^ observed an intrinsic astrocyte calcium spike during stimulation accompanied by a negative BOLD fMRI signal over the cortex in opposite to the evoked neuronal and astrocytic calcium signal positively correlated with the fMRI signal. Tong et al.^24^ increased the number of implanted fibers using a camera approach for detection and was able to demonstrate a different coupling of the two signal modalities for the evoked and the resting paradigm for subcortical regions. Lake et al.^25^ combined fMRI with simultaneously acquired cortex-wide fluorescence calcium imaging and showed that the BOLD response can be predicted by the calcium signal. Van Alst et al.^26^ used the cross-modal approach to investigate the modulating influence of anesthesia on the BOLD signal. Pais-Roldan et al.^27^ used the cross-modal approach to validate pupillometry as another possible contactless way to determine the current brain state in animals, which is well established in humans. Chao et al.^14^ developed a fiber-photometry platform to calculate and validate the hemodynamic response function (HRF) necessary for fMRI analyses due to the fact that the HRF varies for different brain regions in rodents. Ma et al.^16^ found strong evidence that the neural mechanism for resting-state BOLD fMRI depends on the spiking activity of neurons using a cross-modal approach. Zhang et al.^17^ were also able to show that the stimulation-driven BOLD response is significantly affected by the hemodynamic response to independently ongoing brain-wide neuronal activity.

Here, we put forward a framework for extracting the very BOLD response evoked by the SoI: the OPTO-MAgnetic Integration Concept (OPTOMAIC). Combining OPTOMAIC with the fast line-scanning approach,^28^^,^^29^ which samples the BOLD response with a high temporal resolution, also enables high-resolution identification of the spatiotemporal hemodynamics of the extracted averaged BOLD response. For this, the onset of the extracted averaged BOLD response is determined as possibly the best reference value of the underlying SoI,^30^ closing the large temporal gap in the cross-modal approach that exists between the fast optical SoI measurement and the much slower corresponding BOLD response depiction.

## 2 Concepts of Bridging the Temporal and Spatial Gap Separating Optical and Magnetic Resonance Recordings

**Fig. 1:**
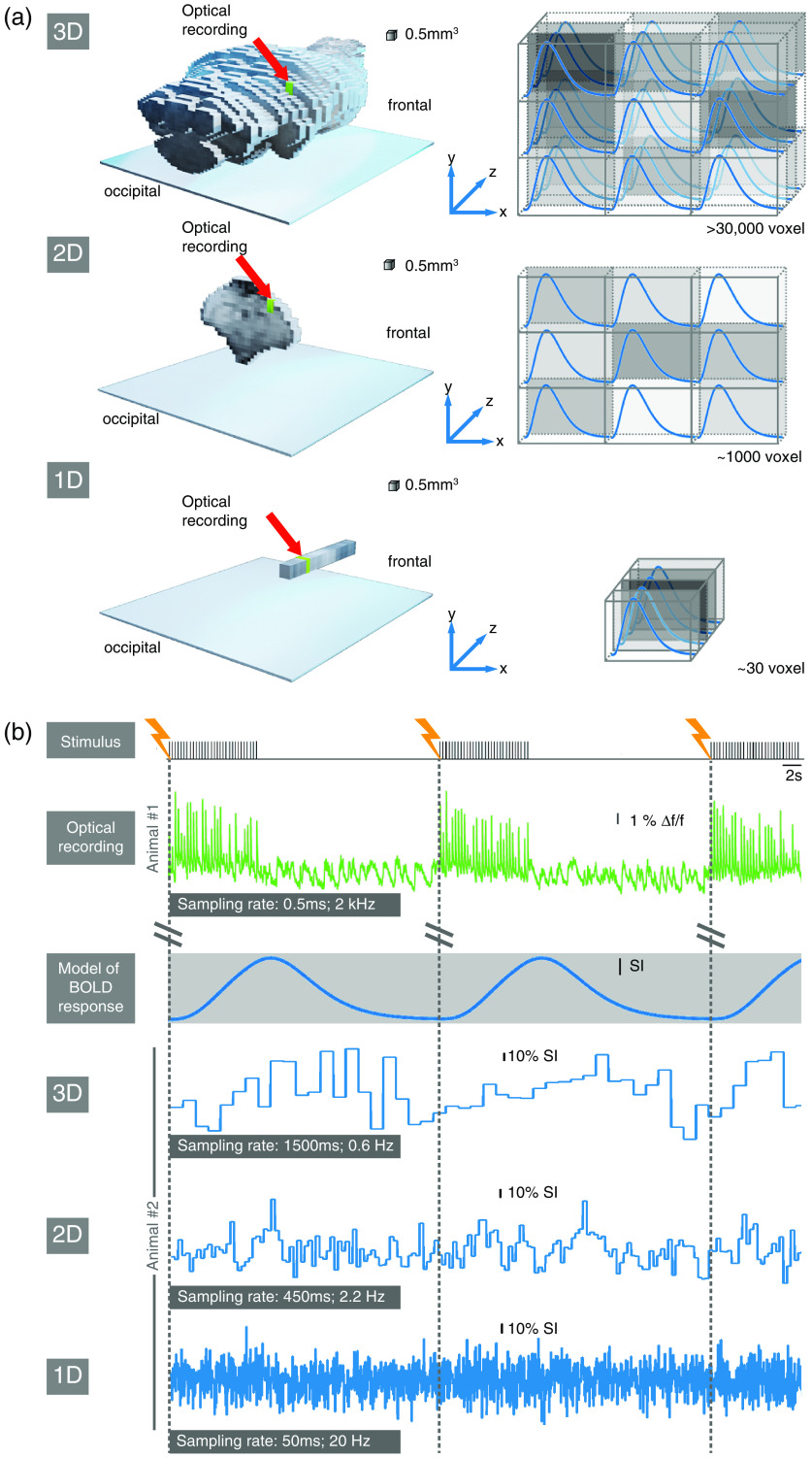
Sampling schemes and corresponding signal traces for different BOLD fMRI approaches in comparison to optical fiber-based calcium recordings. (a) With a 3D BOLD fMRI sampling scheme (upper row) whole-brain coverage is possible, with about 30,000 voxels, resulting in 30,000 independent functional BOLD responses. A 2D single slice sampling scheme reduces the voxel number to around 1000 and a 1D line-scanning approach measures only around 30 voxels. In comparison, optical fiber-based calcium recordings detect only one voxel (in green) in comparable size to a single BOLD fMRI voxel. (b) Matrix of cross-modal signal integration. Forepaw stimuli at 3 Hz for 10 s every 30 s were delivered to the lightly sedated rat in persistent activity brain state resulting in short-latency calcium transients (green: by the courtesy of Schwalm et al.^19^) for each stimulation pulse. In contrast, the expected BOLD response (grayed out) after a delayed onset lasts over the entire stimulation train. However, the expected BOLD response for this stimulation paradigm cannot be identified in the signal traces of the individual presented sampling schemes for individual stimulation trains because the bias due to noise and other hemodynamic processes such as respiration and heartbeat is too strong.

A BOLD fMRI experiment generates spatially defined functional information: the hemodynamic response to a stimulus train or the hemodynamic changes in resting-state are obtained in each recorded voxel. In a preclinical setting in rodents, brain-wide coverage in a range of 30,000 to 100,000 voxels [Fig. 1(a), top row] is possible,^31^^,^^32^ allowing for the identification of brain-wide functional networks comprising of distinct functional nodes.^32^ However, the sampling rate is longer than 1 s, and furthermore, averaging over many neuronal events is required to achieve a sufficient signal-to-noise ratio (SNR) to represent the mean BOLD response for further statistical analysis.

Our goal is to determine the BOLD response onset per voxel very precisely, since this might correlate best with the timing of the putative underlying neuronal event. For this purpose, the sampling rate must be accelerated by reducing the number of imaged voxels in order to obtain a better temporal mapping of the BOLD response and its onset. When moving from 3D imaging to 2D imaging of only a single slice [Fig. 1(a), middle row], the sampling rate can be increased by a factor of approximately 3, while the number of voxels is reduced by a factor of about 30. For 1D line-scanning fMRI [Fig. 1(a), bottom row], the sampling rate is increased again by a factor of ∼9, while the voxel number is again reduced by a factor of about 30.

The following settings were used as an example.

### 2.1 3D

#### 2.1.1 Spatial component

The spatial resolution per voxel of the pulse sequence example for the 3D approach is $(0.32\times 0.29\times 0.8)\;{\mathrm{mm}}^{3}$ and 34 measured slices to obtain whole brain coverage achieving considerably >30,000 measured voxels with a mean BOLD response.

#### 2.1.2 Temporal component

The sampling rate for the completely covered brain volume is 1.5 s or 0.66 Hz.^31^

### 2.2 2D

#### 2.2.1 Spatial component

The spatial resolution per voxel of the pulse sequence example for the 2D approach is $(0.5\times 0.5\times 0.8)\;{\mathrm{mm}}^{3}$ using a 2D-FLASH pulse sequence achieving around 1000 measured voxels with a mean BOLD response.

#### 2.2.2 Temporal component

The sampling rate is around 450 ms or 2.2 Hz per recorded slice.

### 2.3 1D

#### 2.3.1 Spatial component

The spatial resolution per voxel of the pulse sequence example for the 1D line-scanning approach is $(1.0\times 2.0\times 0.3)\;{\mathrm{mm}}^{3}$ resulting in 0.3-mm resolution of the measured line achieving around 30 measured voxels with a mean BOLD response.

#### 2.3.2 Temporal component

The sampling rate per recorded line is 50 ms or 20 Hz.^28^^–^^30^

### 2.4 Cross-modal Opto-magnetic Approach

Thus, the BOLD fMRI experiment is superior to optical fiber-based calcium recordings in terms of the spatial coverage of the brain. The optical fiber-based method cannot be defined as imaging, as only one voxel is functionally recorded, integrating the dynamics of the intracellular calcium concentration of the local neuronal population. The dimensions of this voxel are limited by both the area of staining with the calcium indicator, and the diameter and optical parameters of the optic fibers used, such as the numerical aperture.^6^^,^^19^^,^^33^ Typically, the spatial extend of the voxel using a 200-μm diameter fiber ranges at $(200\times 200\times 300)\;{\mu m}^{3}$.^6^^,^^19^^,^^34^

In contrast, in the representation of neuronal SoI, the optical method is superior to BOLD fMRI. In BOLD fMRI, the measured signal consists of the hemodynamic response to a neuronal event and a strong bias due to signal noise and other non-neuronal hemodynamic processes. Therefore, to reduce this bias, the signal is analyzed over many neuronal events.

The recording of calcium dynamics, on the other hand, is associated with the true SoI as follows: a neuronal action potential leads to the opening of voltage-gated calcium channels, and an elevation of intracellular calcium concentrations, which significantly outlives the duration of a neuronal action potential with a duration of at least 50 ms. Second, the kinetics of the indicator needs to be considered, while the onset of a high-affinity indicator raises with a latency of only a few milliseconds upon an action potential, the off-kinetics do not reflect the actual calcium dynamics. Therefore, it is necessary to record the calcium signal with a high sampling rate for reliable detection of the onsets of the calcium signal as the best correlate of the underlying neuronal action potentials.

We illustrate this using an exemplary setting with a periodic sensory stimulation [Fig. 1(b)]. In an *in vivo* experiment, it is of utmost importance that all raw signal traces are recorded in a joint signal acquisition scheme to ensure temporal synchronicity.^19^^,^^31^

The signal of the optical fiber-based calcium recordings (green) depicts with high temporal resolution the activation of neurons in the recorded volume and thus the neuronal response to each stimulus. Of note, here, we provide an example of a stimulus train, i.e., several stimuli in short succession. Certainly, also single-pulse paradigms are possible, this all depends on the respective neurophysiological question. In this example, we could obtain a short-latency calcium transient upon each stimulus within the stimulation train. To obtain a repeated neuronal response to several stimulus trains, the stimulus paradigm must take into account the neuronal adaptation to the repeated stimuli. This means that the individual stimulus trains must not be too long, and a pause must be planned between the stimulus trains.

Considering the signal traces for each of the presented sampling methods recorded with the same stimulation paradigm measured in another animal, it is noticeable that the sampling rate is considerably accelerated with each reduced spatial sampling dimension. Also, the signal bias due to noise and other hemodynamic processes is so strong that BOLD responses to individual stimulation periods are not identifiable in the measured signal trace. Therefore, preprocessing of the measured signal traces by e.g., filtering and signal averaging over several stimulation periods, to obtain a mean BOLD response, is necessary.

## 3 Employing Optical Neuronal Recordings as a Hypothesis Driven Template with Single Event – Single Voxel Precision in Seven Steps

**Fig. 2:**
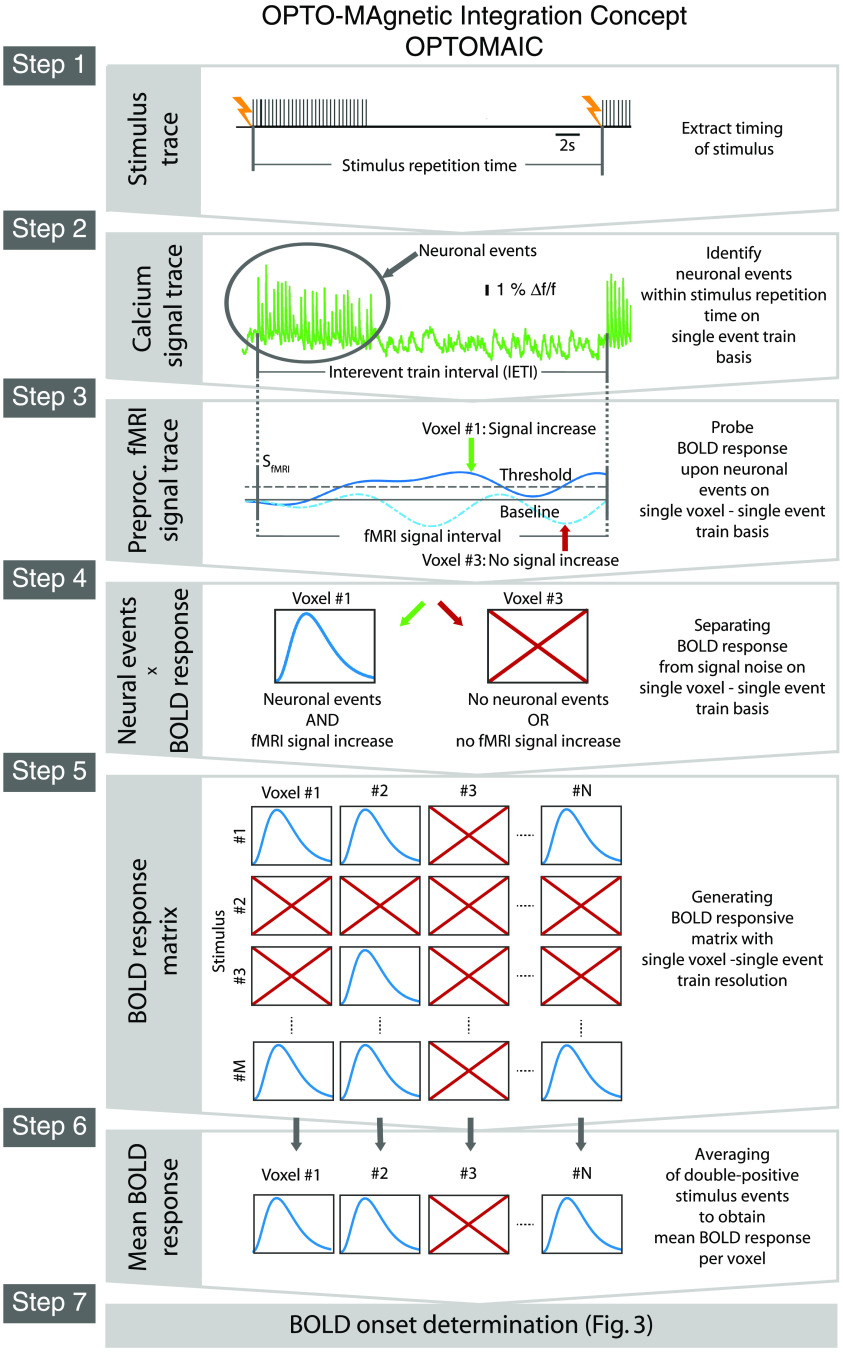
OPTOMAIC as a template for the cross-modal analysis of BOLD fMRI experiment with synchronized optical fiber-based calcium recordings. Starting with the measured calcium signal trace (by the courtesy of Schwalm et al.^19^) identifying the neuronal SoI the mean denoised BOLD response is obtained from the fMRI traces on a single neuronal event – single voxel basis, which is mainly depending on this underlying SoI bridging the spatial gap between both measurement modalities.

OPTOMAIC as a cross-modal analysis approach takes full advantage of the specific strengths of both MRI and optical recordings (please refer also to Fig. 2).

Step 1 Stimulus trace
  From the recorded stimulus trace, the timing of each stimulus train is extracted. Furthermore, the stimulus repetition time between two successive stimulus trains is calculated in which a neuronal event will be considered as a response to the preceding stimulus.
Step 2 Calcium signal trace
  In the calcium signal traces, neuronal events associated with the respective stimulation events are identified as the SoI. The InterEvent Train Interval (IETI) between two consecutive neuronal event trains is also determined.
Step 3 Preprocessed fMRI signal
  The preprocessed fMRI signal trace is probed voxel-wise on a single neuronal event train basis. Only those intervals are retained in which the fMRI signal rises above a threshold value, indicating a putative BOLD response.
Step 4 Neural event x BOLD response
  Now, the cross-modal analysis starts by further selecting those responses which in addition to the putative BOLD response (see Step 3) are accompanied by neuronal events. All other fMRI signal intervals are discarded and not considered in further analysis, thereby significantly reducing the noise bias in the resulting averaged BOLD response.
Step 5 BOLD response matrix
  The BOLD responses measured per voxel and stimulus event train generate the BOLD response matrix. In this matrix, the respective signals of the detected BOLD responses are collected based on single event train and single voxel resolution.
Step 6 Mean BOLD response
  Now it is possible to average all double-positive stimulus events – evoked neuronal events and BOLD response signal increase in voxel considered – for all voxels starting from the BOLD response matrix to generate the denoised mean BOLD response on single voxel basis.
Step 7 BOLD onset determination
  In the last step, the BOLD response onsets are now calculated to obtain the direct dependence of the BOLD response per voxel on the neuronal SoI. For the exact onset determination, there are different approaches, which will be described in more detail in the next paragraph.

## 4 BOLD Onset Determination

The typical duration of a BOLD response upon a neuronal activation event or a train of events in the case of a stimulus train ranges between 5 and 10 s^3^^,^^35^ depending on brain regions. Therefore, the BOLD response for a train of stimulations thus takes correspondingly longer than the stimulation train. With these long durations, how can we at all meaningfully extract the BOLD correlate of fast and short-lived neuronal events? Fortunately, we can, as the long duration rather limits the temporal resolution of differentiation of two successive neuronal events, i.e., the IETI. If two neuronal events arise in temporal succession below 7 s, this will lead to a superposition of their respective BOLD responses. Therefore, in OPTOMAIC we limited the IETIs to intervals longer than 7 s.

However, on a single-event basis, the effective temporal resolution of BOLD fMRI is drastically higher. The pioneering studies of Silva and Koretsky^36^ and Yu,^28^ among others, demonstrated, that the onset of the BOLD response reports the onset of the underlying neuronal event with surprising accuracy: While temporally delayed, the latencies of the BOLD onsets can be very reliable detected, and this allows, e.g., the identification of cortical layer specific temporal succession of neuronal activation. In the template OPTOMAIC put forward here, with cross-modal recordings, we enable the researcher to first, identify all neuronal events occurring in the minimal IETI of 7 s, and subsequently cleanse the BOLD signal trace from the contaminating events, please see above. For the remaining events, a precise calculation or determination of the BOLD onset is absolutely critical to obtain precise spatiotemporal information.

**Fig. 3:**
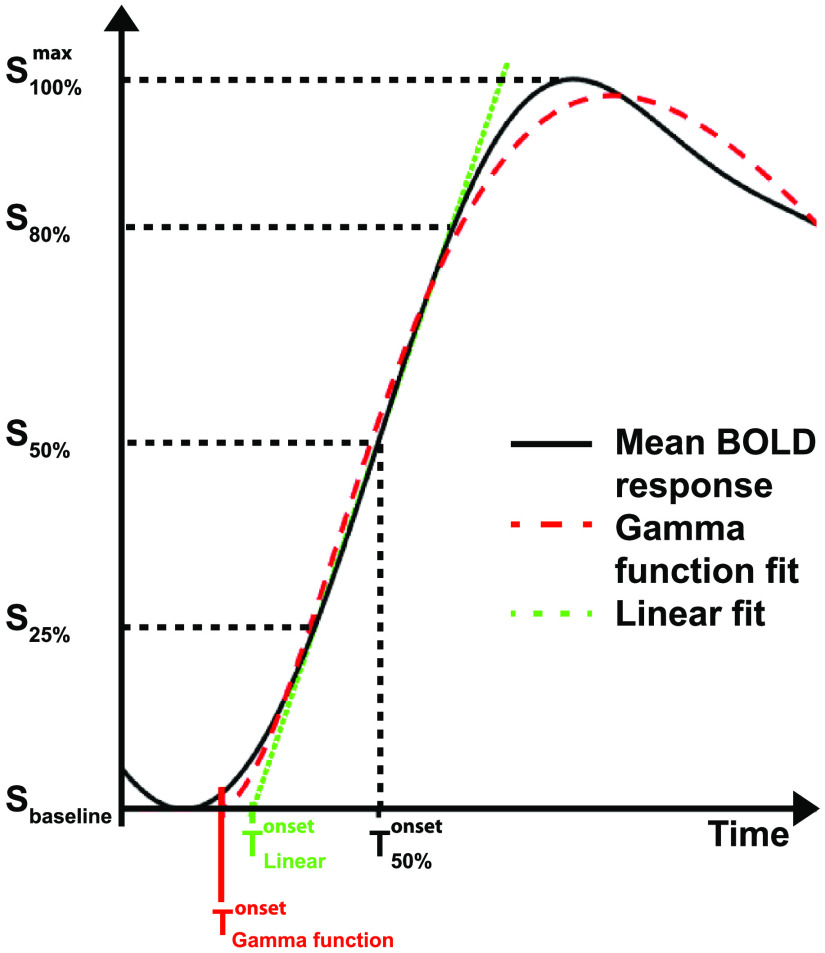
Methods for BOLD response onset determination as the final step of OPTOMAIC enabling further spatiotemporal analyses. The onset of the mean BOLD response on single voxel basis can be calculated by three different approaches: (1) The timepoint when the mean BOLD response rises to 50% of its signal peak (black); (2) The intercept of the linear fit to the rising slope of the mean BOLD response with the baseline signal defined as the mean of the time interval of 1 s prior to stimulation (green); and (3) The first nonzero value of a fitted HRF (red).

To minimize bias by noise we suggest three different onset parameters for BOLD response onset determination described by Yu et al.^30^ and Albers et al.:^29^ $T_{50}$, $T_{\mathrm{lin}}$, and $T_{0}$, defined as follows (please refer also to Fig. 3):

### 4.1 Onset Determination Based on Percentage Signal of Maximum Peak

$T_{50}$ is the time to half maximum signal of the mean BOLD response used by Albers et al.^29^ Also, $T_{10}$ of 10% to maximum signal is a common value avoiding contamination by noise. Another onset time is given by the initial slope of the BOLD response reaching baseline signal plus two times of standard deviation of the baseline signal before the stimulus.^30^

### 4.2 Linear Fit on the Initial Rise

$T_{\mathrm{lin}}$ is the intercept with the baseline signal of a linear fit to the rising signal slope of the mean BOLD response between the first datapoints reaching 25% and 80% of the signal maximum. The baseline signal is defined as the mean of the time interval of 1 s before the stimulation.^29^

### 4.3 Fitting of a Function

$T_{0}$ is the first nonzero value of a fitted HRF model by Boynton et al.^37^ to the mean BOLD response, expanded by a parameter allowing for variable amplitudes

$$h(t)=A*\frac{{\left(t-T_{0}\right)}^{a-1}*b^{a}}{\text{gamma}(a)}*e^{-{\left(t-T_{0}\right)}^{b}}\;\text{for}\;t\geq T_{0}$$

$$h(t)=0\;\text{for}\;t<T_{0}$$

The amplitude A, $T_{0}$, and the rate parameter b, representing the peak width has to be adapted within the parameter limits A=[0,1], $T_{0}=[0.4,\;3.\;5]\;s$, b=[0.5,3]  1/s, whereas the shape parameter a=3 is set as constant.^29^

## 5 Technical Implementations

The description of the technical implementation is divided into three subsections.

In the first section, the step-by-step procedure for preparing the experimental animal for optical fiber-based calcium recordings is explained consisting of the injection of a fluorescent dye to visualize cellular calcium fluctuations followed by implantation of the optical glass fiber.

The second section deals with the cross-modal measurement on the MR system. The experimental setup is described, and the parameters for the fast line-scanning fMRI technique are given.

In the third section, the implementation of OPTOMAIC as a hypothesis-driven single event – single voxel mean BOLD response determination approach with subsequent BOLD response onset calculation is explained in more detail.

### 5.1 Optical Fiber-Based Calcium Recordings

**Fig. 4:**
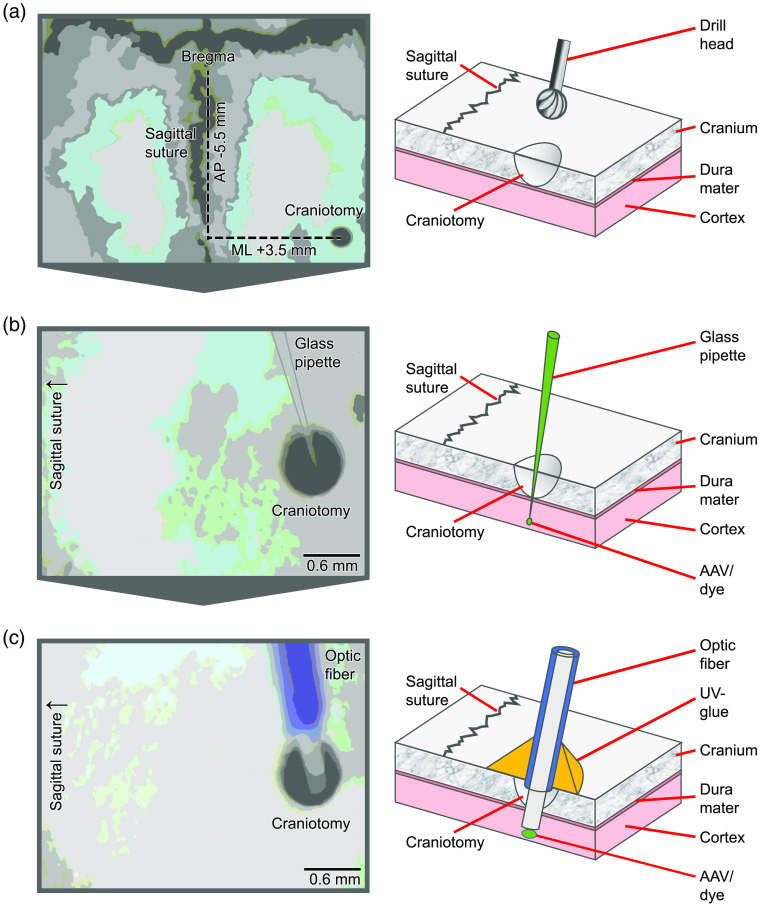
The procedure is shown for the visual cortex of a rat, V1; (a) The skin and periosteum are removed, skull bone is cleaned, and bone junctions Bregma and Lambda are visible. With a diamond drill head a craniotomy is performed in the area in which the optical fiber will later be implanted. (b) The calcium dye is tip-loaded into a glass pipette and injected stereotactically piercing the dura mater. (c) The prepared optical fiber is also implanted stereotactically. For better stability during the consecutive cross-modal experiment, the coating of the fiber is removed only to the extent that part of the coating can be pushed into the drill hole without implanting the fiber too deeply. The fiber is then bonded to the skull with UV adhesive which is piled up around the fiber in a pyramid shape to achieve higher stability.

The step-by-step instructions for injecting a calcium dye, such as Oregon Green 488 BAPTA-1 (OGB-1), and optical fiber implantation into the brain of rodents have been described in detail elsewhere^5^^,^^8^^,^^19^. Here we will only give a brief outline with special attention to the implications arising from the cross-modal measurement consisting of BOLD fMRI combined with simultaneous fiber-based calcium recordings and how to solve them (refer also to Fig. 4).

First, the BOLD fMRI measurement demands some modification due to its high magnetic field environment and its signal generation. For example, the ointment to prevent dry eyes must be water-based, otherwise signal artifacts will appear in the fMRI images. Also, an inhalation anesthetic based on isoflurane is recommended for the surgeries, because of its easy dosing and its use in MR experiments as a standard anesthetic. Therefore, analgesia, e.g., based on buprenorphine, must also be administered from the start.

For the craniotomy a ceramic or diamond drill head should be used to avoid metal shavings in the drilled hole creating signal artifacts in the following BOLD fMRI measurement. The opening of the brain must be performed very carefully avoiding bleedings due to injury of the dura mater, which otherwise also generates signal artifacts in the fMRI measurement.

After the slow and careful injection of the calcium dye and a sufficient pause for incorporating the dye into the neurons the optical fiber implantation must be performed. For this purpose, the optical fiber must be passed through the lead-through of the MR acquisition coil before its stereotactic implantation to prevent subsequent disconnection and reconnection of the fiber to the calcium recording equipment. After optical fiber implantation, the signal quality of the calcium signal must be probed by a test measurement and, if necessary, the implanted depth must be corrected before the optical fiber is glued to the skull with a UV glue, because the fiber-based calcium signal may be contaminated by hemodynamic processes if the fiber is positioned, e.g., directly near a large blood vessel.^38^ However, a hemodynamic contamination may be most problematic in widefield calcium imaging approaches as described in Valley et al.^39^ An advantage of optical fiber-based calcium recordings represents the on-the-fly assessment of the data quality, i.e., the identification of the neuronal SoI, while implanting the fiber. We recommend performing short test measurements with appropriate stimulation while the fiber is lowered into the brain, before and after the fiber is glued to the skull. We stop the insertion of the fiber, once we identify the neuronal SoI, and even carefully and slowly change the x−y position of the fiber, which is still possible when recording from superficial cortical structures. This approach increases the success rate significantly. Yet, certainly, it cannot be avoided that a rupture of a hidden blood vessel occurs, in which case the respective experiment cannot be continued (please refer also to Refs. 5 and 38).

During subsequent bonding of the fiber to the skull, the adhesive is piled up on the optical fiber in a pyramid-like manner to achieve greater stability of the implanted optical fiber. After this procedure, a test measurement is again recommended.

Then the fMRI measurement can be prepared by fixing the animal still in anesthesia on the MR animal bed with the acquisition coil on its head, installing the biomonitoring modules, and positioning the animal in the MR system.

For longitudinal measurements, and for the subsequent use of optical fiber-based calcium recordings in combination with behavior, it might be advantageous to implant a fiber stump only. This allows for attaching the recording optical fiber by means of a commutator. However, two important limitations need to be mentioned in the context of cross-modal opto-magnetic recordings: first, common commutators and implantable fiber stumps contain ferromagnetic material, rendering applications in this context obsolete. There are ceramic commutators; however, they tend to be expensive and are rather fragile. Second, each commutator introduces loss of signal due to light diffraction at the interface between the optical fibers. While in optogenetics experiments, this is less of a concern, as loss of excitation light can be compensated by increasing light intensity, even more so when using laser light sources, yet, for optical fiber-based calcium recordings, the intensities of the calcium indicator fluorescence range in nW, so that any loss of light inevitably decreases the SNR up to the point of the inability to identify the underlying neuronal SoI.

### 5.2 Signal Integration, including fMRI Signal, Optical Signal, and Biomonitoring

**Fig. 5:**
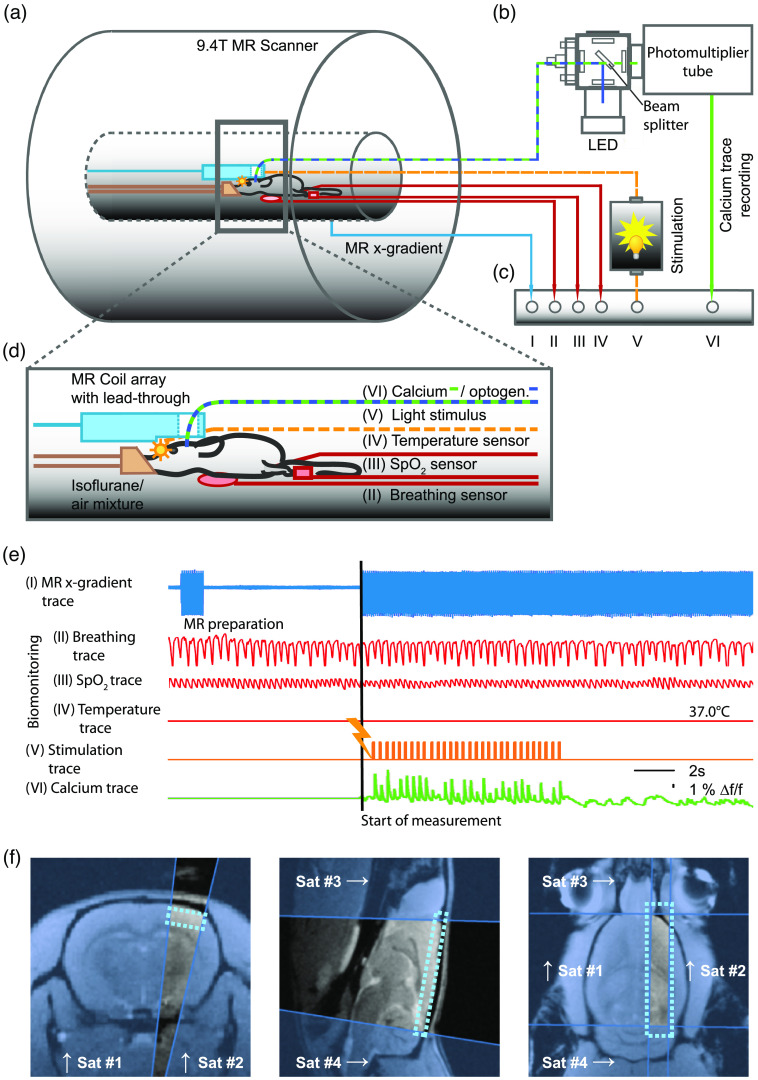
Experimental setup of the cross-modal line-scanning fMRI measurement with simultaneous optical fiber-based calcium recordings. (a) The animal is placed inside the bore of the MR system together with the monitoring sensors, the optical fiber for the visual stimulation paradigm, and the optical fiber for calcium signal trace recordings (b) Optical fiber calcium recordings setup consisting of an LED for excitation of the calcium dye emitting blue light and a unit with photomultiplier tube detecting the green fluorescence signal containing the functional neuronal SoI. (c) The detected calcium signal, the sensory stimulation signal, the switching of the MR gradients as well as the biomonitoring traces are digitized and stored simultaneously using an electronic device. (d) Magnified view of the setup inside the MR system. The optical fiber (green blue dashed) for calcium recordings is passing through the lead-through of the MR acquisition coil array (light blue) into the brain of the animal. For light pulse stimulation, an additional fiber (orange) is placed nearby the eyes of the animal. As biomonitoring signals temperature, ${\mathrm{SpO}}_{2}$, and breathing traces (red) are collected. (e) As an example, an interval of all recorded signals is depicted. Of note, all signal traces must be synchronously measured. The switching of the MR gradient is used in the analysis for temporal synchronization of the line-scanning fMRI traces with the calcium traces (by the courtesy of Schwalm et al.^19^). (f) Localizer images (axial, sagittal, and coronal) show the position of the measured line-scanning fMRI signal line together with four additional saturation pulses for limiting the FOV in the brain of a rat.

An electronical device (Cambridge Electronic Design Limited, Cambridge, England) records all electronic signals consisting of the vital parameter traces, the depiction of the MR gradient switching for synchronization of the signals recorded by each modality, and the calcium traces, runs the optical fiber-based calcium recording setup, and provides the stimulation paradigm, if necessary (depicted in Fig. 5). The advantage of this device is that all signals are recorded and applied with the same time resolution and are thus synchronized with high temporal resolution and digitized for later analysis.

### 5.3 fMRI Sequences

#### 5.3.1 3D

The pulse sequence commonly used nowadays for 3D fMRI measurements of rat brains is based on a single-shot gradient-echo excitation with a fast EPI readout. The gradient echo is measured as it has a high dependence on the $T_{2}^{*}$ contrast and thus shows the BOLD contrast more clearly than the spin echo, which generates a weaker and more localized BOLD signal due to its $T_{2}$ dependence. The gradient echo is coupled with the fast acquisition mode of the EPI readout, in which a whole slice can be imaged per excitation. Thus, up to 34 individual slices with whole-brain coverage can be recorded within the repetition time of 1.5 s. An exemplary single-shot gradient-echo EPI sequence has the following parameters^31^: a voxel size of $(320\times 290\times 800)\;{\mu m}^{3}$, TE=14.5  ms to obtain a sufficient $T_{2}^{*}$ signal contrast, and FA=65  deg. To avoid signal artifacts a fat suppression pulse and a regional saturation pulse suppressing signal of the jaw region are switched once at the beginning of each volume recording within the TR.

#### 5.3.2 2D

A 2D slice can be achieved with different signal recording schemes of the K-space representing the spatial frequencies, from which the well-known grayscale image is generated using the Fourier transform. In addition to line-by-line Cartesian K-space sampling, non-Cartesian schemes such as radial or spiral sampling are also possible, which can lead to a significant reduction in the acquisition time for a single slice but are complicated in image reconstruction due to easily introduced distortions and image artifacts and are not yet available as a standard for fMRI. As an exemplary pulse sequence for 2D fMRI, the parameters for a spoiled FLASH gradient echo sequence are given here achieving a voxel resolution of 0.5×0.5×1.0  mm with (TR/TE)=(450/18)  ms achieving sufficient signal for fMRI analysis.

#### 5.3.3 1D

The used 1D line-scanning sequence^28^^,^^29^ is based on a spoiled FLASH gradient echo sequence with removed phase encoding steps. Therefore, the field of view (FOV) of the measured signal must be restricted additionally by four regional saturation pulses using relatively long sinc7H pulses to get a pencil-shaped signal “line” with efficient saturation restricting of the FOV width to around 2 mm and resulting in a rather long repetition rate of TR=50  ms for each line recorded [as depicted in Fig. 5(f)]. In reconstruction, the Fourier Transformation should be applied only in readout direction to obtain corresponding gray-scale signal lines over time. The other parameter settings are as follows: TE=18  ms to achieve a $T_{2}^{*}$ dependency for proper BOLD response detection, α=13  deg, the overall measurement time should be around TA=20  min to measure enough neuronal events for BOLD onset analysis.

### 5.4 Implementation of OPTOMAIC

**Fig. 6:**
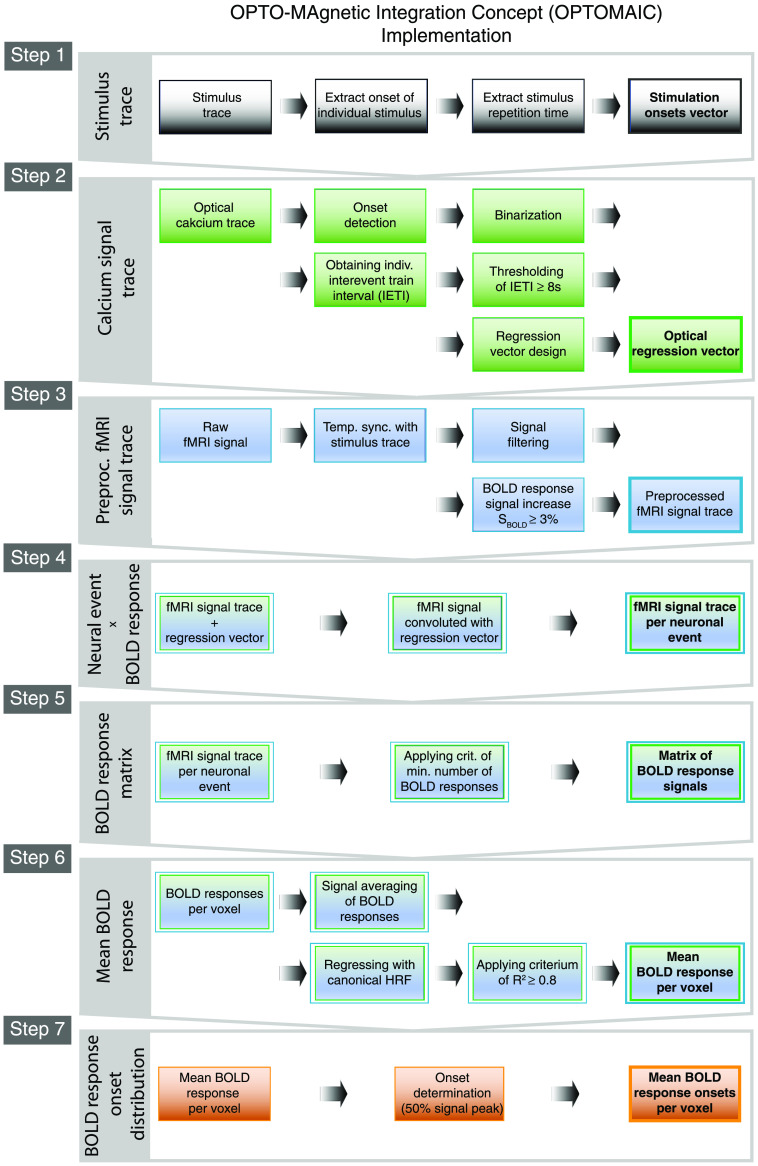
Step-by-step instructions for implementing the analysis template OPTOMAIC for cross-modal BOLD fMRI measurements with synchronous optical fiber-based calcium recordings. The extraction of the SoI from the calcium recordings is explained, as well as the intertwined analysis steps to generate the unbiased mean BOLD response from the fMRI signal trace on single neuronal event – single voxel basis solely representing the SoI voxel-wise. Of note, each step commences with the distinctive source data followed by several processing steps and resulting in a new outcome data set (in bold) to obtain the averaged BOLD response onset per voxel, in the end, enabling further spatiotemporal analysis.

The template OPTOMAIC for intertwined analysis of the cross-modal BOLD fMRI measurements with simultaneous optical fiber-based calcium recordings presented in Figs. 2 and 3 and described in paragraphs 3 and 4 will now be discussed in more detail regarding its implementation (please refer also to Fig. 6). For a better understanding of the following text, it is necessary to remember that the individual analysis steps build on each other, as already mentioned in paragraph 3.

For the analysis of resting-state fMRI data combined with optical fiber-based calcium recordings the step 1 of OPTOMAIC is obsolete and the analysis starts with step 2 processing the calcium traces.

Step 1: Extracting timing of individual stimulation events from the recorded stimulus trace

In the first analysis step, the individual stimulation events are identified in the recorded stimulus trace so that the corresponding stimulated neuronal events can be determined in the calcium signal in the next step.

Starting with the stimulus trace, both the onset for each individual stimulation event and the repetition time to the subsequent stimulation event is determined, whereby a stimulation event can consist of a single stimulation or a complete stimulation train, as in the example in Fig. 2. Subsequently, a stimulus vector is generated by binarization, which is one for a stimulus event onset and zero otherwise. The generated stimulus vector has the same sampling rate as the calcium signal trace to synchronize the stimulus vector and the calcium signal trace.

As a result of this step, we get the stimulus event onset vector.

Step 2: Generation of the optical regression vector from the calcium signal trace representing the underlying SoI

In this analysis step the neuronal SoI consisting of consecutive evoked neuronal events is identified and prepared as a regression vector for further cross-modal analysis.

For this, we first synchronize the recorded calcium signal with the stimulus event onset vector. Then, the evoked neuronal events in the calcium signal are identified and their respective onsets are determined.

The identification of neuronal events in calcium traces represents a critical step in the analysis pipeline of optical neuronal recordings and optical imaging. We already put forward two publications on this topic: Fu et al. and Guimaraes-Backhaus et al.,^8^^,^^40^ as ultimately, optical recordings do also just represent a proxy for the underlying electrical activity. Yet, the identification of the putative neuronal event, or better said its signature in the calcium trace, depends very much on the neurophysiological nature of the event, and cannot be generalized. E.g., a visual evoked potential (please see our recent publication: Zaer et al.^41^) is fundamentally different to a slow oscillation (please see Ilhan-Bayrakci et al.^42^), requiring differential analyses routines.

Again, a neuronal event can consist of a single neuronal activation due to a single stimulus or a neuronal activation train, as presented in Fig. 2. It is important to note that each individual evoked neuronal activation has a short latency to each individual stimulus by which it is generated independent if it is a single stimulus or a stimulus train. Therefore, the individual evoked neuronal activation must appear within a short range of latencies after the stimulus that generates it. All other neuronal activations not related to a stimulus most likely arise spontaneously and are not considered further, because they are not representing the detected SoI.

The onsets of the identified evoked neuronal events are also binarized with one for onset and zero otherwise. In addition, for each neuronal event, the IETI to the previous neuronal event is determined.

The following criteria must be fulfilled for neuronal events in the further analysis, otherwise they are discarded:

• The individual neuronal events must have been generated by stimulation.
• The respective IETI to the preceding neuronal event must be >7  s to prevent bias due to overlapping BOLD responses.

The onsets that meet these conditions are combined into a so-called optical regression vector that has the same temporal resolution as the fMRI measurement, for a line-scanning fMRI experiment, this is a down sampling from 2 kHz to 20 Hz.

As a result of this analysis step, we obtain the optical regression vector, which consists of ones for the selected onsets of evoked neuronal events and zeros otherwise.

OPTOMAIC can also be easily used to analyze resting-state fMRI with optical fiber-based calcium recordings. In this case, the first step regarding the stimulation train has to be skipped and the analysis starts with step 2. The optically recorded spontaneous neuronal activation events generate the SoI and are therefore taken into account for further analysis, where the criteria for neuronal events are only adjusted to an IETI>7  s, where IETI is the interval between successive spontaneous neuronal events.

Step 3: Preprocessing of the fMRI signal trace per voxel

In step 3 of the implementation of OPTOMAIC the preprocessing of the fMRI signal trace is performed voxel-wise before it is linked in step 4 to the optical regression vector resulting from the analyzed calcium signal. The preprocessing consists of three consecutive substeps: first, the measured fMRI signal trace is temporally synchronized with the stimulus trace. Then, the signal is filtered with a low-pass filter using a MATLAB routine that performs zero-phase digital filtering (filtfilt) with a cut-off frequency of 0.3 Hz to minimize the strong bias due to signal noise. At this point, other filtering methods adapted to the individual experimental question can be inserted.

Third, for each stimulus interval, it is examined whether there is a signal increase of at least 3% in the time interval 3 to 6.5 s after the stimulus compared to the baseline signal. As baseline signal, the mean fMRI signal in the time interval of 1 s before the stimulus is taken.

The result of this analysis step is then the preprocessed fMRI signal trace with minimized bias of noise and other non-neuronal hemodynamic signal influences on a single voxel basis.

Step 4: Cross-modal linkage of preprocessed fMRI signal trace with identified neuronal events representing the neuronal SoI on a single voxel basis

Here, we intertwine on a single voxel basis both the preprocessed fMRI signal trace and the neuronal SoI to obtain the fMRI signal, which is predominantly dependent on the neuronal SoI.

For this, we mathematically convolute the preprocessed fMRI signal trace and the optical regression vector.

Thus, as a result the fMRI signal on single event - single voxel basis is obtained.

Step 5: Generation of the BOLD response matrix on single neuronal event – single voxel basis

The BOLD response matrix collects all identified BOLD responses on single neuronal event train – single voxel basis. On single voxel basis also the criterion of a minimum number of 20 identified BOLD responses has to be fulfilled as a validity check. The respective value is given as a starting point and must be tailored to the respective experimental condition.

The result of this analysis step is the BOLD response matrix consisting of all identified BOLD responses on single neuronal event – single voxel basis.

Step 6: Calculation of the mean BOLD response per voxel

In this analysis step, the mean BOLD response is calculated for each voxel, which is cleaned of signal bias and evoked at least in part by the measured neuronal SoI and thus represents its hemodynamic signature in each considered voxel.

For this, we start with combining of all identified BOLD responses per voxel obtaining a mean BOLD response, which must also meet an additional criterium as a validity check, again, the respective values are given as a starting point:

The shape of each mean BOLD response is probed for its similarity to the canonical BOLD response model. For this purpose, the correlation value in the regression analysis must be high enough to sort out measured mean BOLD responses that are corrupted by partial volume effects. We found that a value of $R^{2}\geq 0.8$ might be sufficient as a criterion for the shape of the mean BOLD response.

As a result of this analysis step, the local distribution of mean BOLD responses per voxel is finally obtained representing the hemodynamic signature of the underlying SoI.

Step 7: Voxel-wise BOLD response onset determination

In this last analysis step of OPTOMAIC the onset of the measured mean BOLD response is calculated voxel-wise for further spatiotemporal analysis of the hemodynamic BOLD response signature of the underlying neuronal SoI, which is not the subject of this publication.

Starting from the mean BOLD response per voxel calculated in step 6, which depends at least in part on the neuronal SoI, the corresponding BOLD response onset is calculated using the methods presented in paragraph 4, e.g., as the time at which the signal rises to 50% of the signal peak.

As a result of this analysis step and of OPTOMAIC, the local distribution of the onsets of the measured mean BOLD responses is obtained on a single voxel basis.

### 5.5 Application of OPTOMAIC on a Sample Data Set

**Fig. 7:**
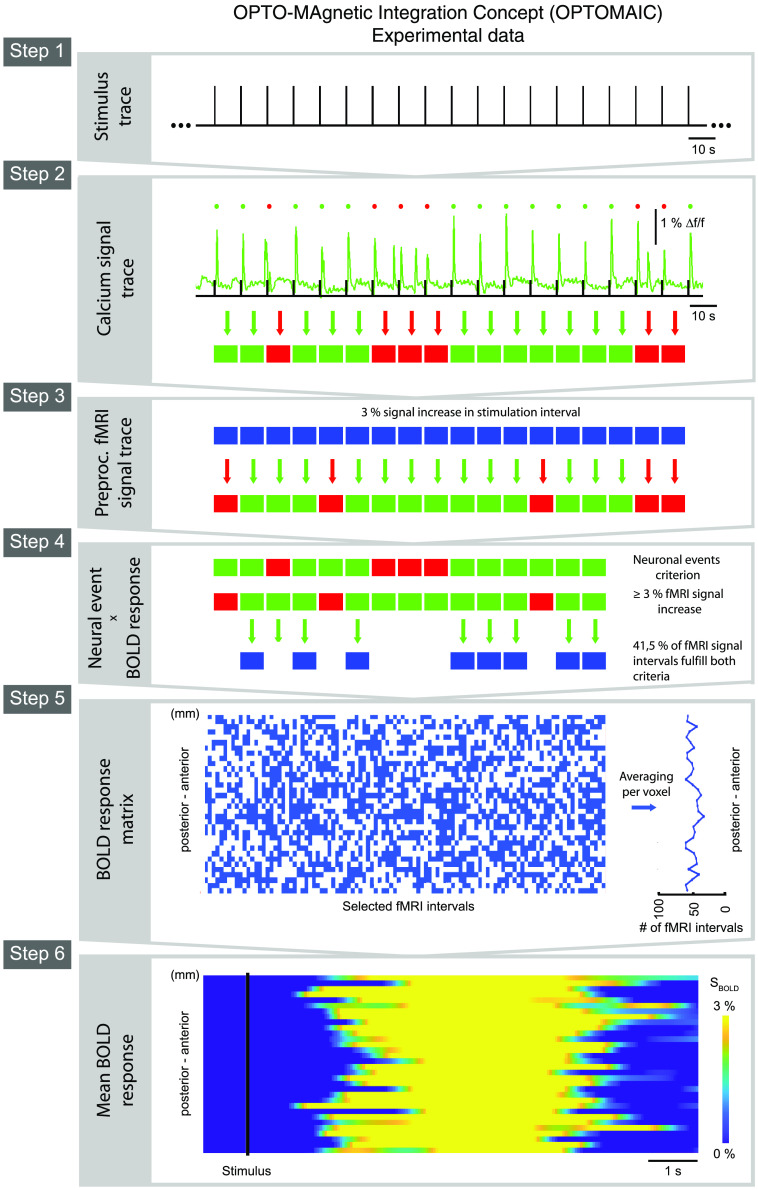
Application of OPTOMAIC to an experimental cross-modal data set. For 125 visual stimulations at 10 s intervals, the associated calcium trace in the visual cortex and the line-scanning fMRI traces of 34 voxels recorded in the cortex from anterior to posterior are evaluated in the brain state of slow oscillation-associated slow-wave activity with pancortical BOLD response.^19^ As exemplified in step 2 of OPTOMAIC, 84% of the visual stimulations evoked exactly one neuronal event and are considered in further analysis of the fMRI traces. In step 4, 41.5% of the stimulations with a neuronal event and a signal increase of at least 3% are identified across all voxels and shown in blue in the BOLD response matrix in step 5. The respective mean BOLD responses from anterior to posterior are calculated per voxel depicted in step 6 showing the expected pancortical hemodynamic response to the stimulus.

The individual steps of the OPTOMAIC analysis are shown on a cross-modal data set measured *in vivo* (refer to Fig. 7). The measurement took place under isoflurane anesthesia in the slow oscillation associated slow-wave activity brain state^6^^,^^19^^,^^31^ in a female Lewis rat (∼220  g). Both the fluorescence signal of OGB-1 in the visual cortex as an optical calcium signal and the line-scanning fMRI signal of a line placed in posterior–anterior axis along with the cortex measuring 34 voxels were recorded simultaneously for 128 visual stimulation intervals produced by a flashlight of 10 ms duration every 10 s delivered to both eyes. The overall measurement time was 21 min 20 s.

Step 1: Due to signal settling in the fMRI measurement and synchronization of the fMRI signal with the optical calcium trace 125 stimulation intervals were considered for further analysis.
Step 2: The onset detection of neuronal slow wave events in the calcium trace is based on the algorithm initially proposed by Seamari et al.^43^ and applied to cross-modal analysis in two recent publications of ours.^19^^,^^31^ It evaluates the crossover of moving averages with a long and a slightly shorter windowing. The crossing point can be taken as a good approximation for an onset or offset if the slope momentum of the measured signal is appropriate.

The detected slow wave events are sorted into evoked and spontaneous slow waves. Only those visually evoked slow waves are retained which were neither preceded in the stimulation interval before nor followed within a 7.5 s time interval by a spontaneous slow wave. 84% of the stimulation intervals met this criterion for neuronal events and were used for further analysis.

Step 3: The fMRI data were low-pass filtered at 0.3 Hz, and the mean signal rise in the interval 3 to 6.5 s after stimulus application was tested for the 3% signal increase criterion for each measured fMRI signal interval.
Step 4: 41.5% of all fMRI signal intervals met the neuronal event AND the 3% signal increase criteria and were considered for the construction of the BOLD response matrix.
Step 5: The BOLD response matrix spans over 34 voxels and 105 selected stimulation intervals. To calculate the mean BOLD response in each voxel, we averaged over ∼50 neuronal events exhibiting a signal increase of at least 3% per voxel.
Step 6: The mean BOLD response shows a pancortical evoked hemodynamic response to the stimulus train as proposed by Schwalm et al.^19^ for these measurement conditions of the slow oscillation associated slow-wave brain state.

**Fig. 8:**
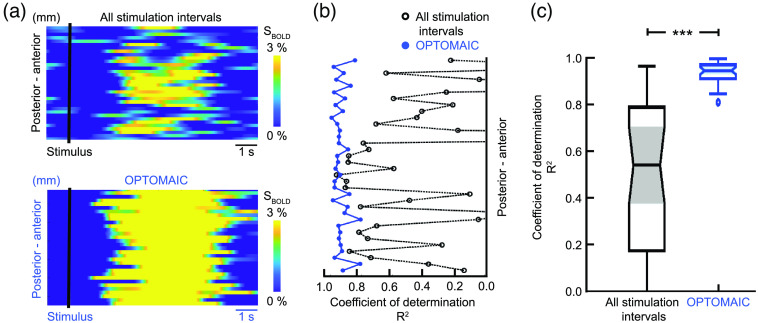
In an experimental dataset, OPTOMAIC significantly outperformed the classical analysis scheme. Comparison between OPTOMAIC-calculated mean BOLD responses per voxel and responses averaged over all stimulation intervals is performed for an experimental dataset in the slow oscillation-associated slow-wave activity brain state. (a) The mean BOLD responses averaged over all stimulation intervals (black) show a cortex-wide noisy signal increase, whereas the pancortical mean BOLD response after analysis with OPTOMAIC (blue) is clearly visible for all voxels in the field of view confirming the findings of Schwalm et al.^19^ (b) The coefficients of determination $R^{2}$ were calculated as a measure for the goodness of fit of the mean BOLD responses to the postulated BOLD response function.^37^ The $R^{2}$-values of the approach averaging over all stimulation events (black) show a high fluctuation meaning that the comparability of the experimental signal traces with the postulated BOLD function is not given. In contrast, the mean BOLD responses analyzed with OPTOMAIC (blue) show a high correlation with the theoretical BOLD function across all voxels indicating the successful extraction of the BOLD response evoked by the neuronal SoI. c) The non-parametric statistical Kruskal–Wallis test indicates a highly significant difference (p<0.001) between the $R^{2}$ values for the OPTOMAIC analysis and the analysis averaging the fMRI signal over all stimulation intervals.

To illustrate the quality of the OPTOMAIC analysis, the mean BOLD response of OPTOMAIC was compared with the analysis averaging over all stimulation intervals (Fig. 8). While the fMRI signal averaged over all stimulation intervals suggests a spatially patchy BOLD response, the mean BOLD response of the OPTOMAIC analysis shows a clear pancortical extension. The quality of the respective mean BOLD response signals obtained by both analysis methods per voxel was investigated using the fit to the postulated BOLD response^37^ and the coefficient of determination $R^{2}$ was further considered as a measure for the goodness of fit. While the coefficient of determination $R^{2}$ for the analysis averaging over all stimulation intervals is highly scattered for different voxels, the $R^{2}$ values for all voxels of the OPTOMAIC analysis is above 0.8, indicating the reliable extraction of the extended BOLD response from the measured line-scanning fMRI signal.

## 6 Outlook

Optical imaging of neuronal activity using proxies of neuronal action potentials has developed at an astounding pace in the last decade. Currently, fluorescent indicators changing their fluorescence upon binding of calcium are most widespread, due to the following two reasons: First, current imaging techniques, such as two-photon imaging do not allow for a fast repetition time, at least not for full-field imaging. Typically, using a resonant scanner, the temporal resolution ranges at 30 Hz. Consequently, the duration of the optical signal needs to exceed 30 ms to allow for detection with two-photon imaging methods. The elevation of intracellular calcium upon the opening of voltage-gated calcium channels provides for a temporal extension of the SoI, albeit at the costs of not being able to resolve a fast action potential train. Second, the SNR of fluorescent calcium indicators, either synthetic or genetically encoded, exceeds current indicators of other ions, such as sodium. What is more, the intrinsic kinetics of the indicator needs to be considered, leading to a further prolongation of the SoI, particularly concerning the off-kinetics. Consequently, even though optical fiber-based calcium recordings exhibit a temporal resolution of several kHz, the effective temporal resolution is much smaller. This has an important implication on the temporal gap between optical and magnetic methods: Clearly, the typical BOLD response ranges around 5 to 10 s, still significantly longer than the typical calcium transient upon a single action potential, ranging at 100 ms,^40^ yet only being separated by two order of magnitudes, compared to three orders of magnitude when comparing the duration of a neuronal action potential of about 3 ms. What is more, while the long duration of the BOLD response limits resolving the correlate of two SoI with interevent train intervals below 7 s, the onset of the BOLD response provides a separate, and temporally rather highly defined readout. Consequently, applying fast fMRI techniques such as the above-mentioned line-scanning method, bringing optical and magnetic recording in a similar range in terms of effective resolution.

The main conceptual advance of OPTOMAIC represents the concept of steps building upon each other. Traditionally, a task-based BOLD fMRI signal analysis averages over all measured trials. Even though sometimes individual trials are being discarded, e.g., due to the change in the physiological state of the animal such as the breathing rate, there is no trial selection based on the neuronal response upon each trial. Here, we put forward the use of the simultaneously acquired neuronal SoI for the trial selection, thereby taking full advantage of the higher sensitivity of optical recordings. This is conceptually different from merely correlating both signals. While there are rare examples of stereotypical and reliable neuronal response patterns, most neuronal SoIs occur with a given response probability, highly dependent not only on the internal and external state of the animal and the underlying brain circuits. By cleansing the averaged BOLD response from the upon-responders, and from neuronal events occurring faster than the duration of BOLD response time, we achieve a much more refined BOLD response pattern, increasing the fraction of the BOLD response which actually reflects the underlying neuronal SoI. What is more, by constructing a regression vector based on the optical SoI, one can convert a task-free paradigm into a task-driven paradigm, with the SoI as task. Of note, this concept is not limited to intertwining optical and magnetic methods applicable in preclinical research only but can be applied in cross-modal approaches amenable in clinical settings. Indeed, we just provide evidence, that a regressor build upon neuronal SoI in EEG recordings may yield a rather specific BOLD signature.^42^ We strongly suggest that concepts uniting the specific strengths of the respective methods in cross-modal approaches rather than a one-size-fits-all method may advance the field.

## Code Availability

The code of OPTOMAIC written in MATLAB (The MathWorks Inc.) is available on GitHub: https://github.com/Strohlab/OPTOMAIC_Neurophotonics2022.
